# Efficient Enzyme-Free Biomimetic Sensors for Natural Phenol Detection

**DOI:** 10.3390/molecules21081060

**Published:** 2016-08-13

**Authors:** Luane Ferreira Garcia, Aparecido Ribeiro Souza, Germán Sanz Lobón, Wallans Torres Pio dos Santos, Morgana Fernandes Alecrim, Mariângela Fontes Santiago, Rafael Luque Álvarez de Sotomayor, Eric de Souza Gil

**Affiliations:** 1 Faculdade de Farmácia, Universidade Federal de Goiás, Goiânia, GO 74690-970, Brazil; luane.fg@hotmail.com (L.F.G.); morgana.alecrim@hotmail.com (M.F.A.); mariangelafs@gmail.com (M.F.S.); 2 Instituto de Química, Universidade Federal de Goiás, Goiânia, GO 74690-970, Brazil; desouzaar@gmail.com (A.R.S.); manger84@gmail.com (G.S.L.); 3 Departamento de Farmácia, Universidade Federal dos Vales do Jequitinhonha e Mucuri (UFVJM), Diamantina, MG 74690-970, Brazil; wallanst@yahoo.com.br; 4 Departamento de Química Orgánica, Universidad de Cordoba, Cordoba 14014, Spain; q62alsor@uco.es

**Keywords:** metal oxides, modified electrodes, enzyme-free biosensors, rutin

## Abstract

The development of sensors and biosensors based on copper enzymes and/or copper oxides for phenol sensing is disclosed in this work. The electrochemical properties were studied by cyclic and differential pulse voltammetry using standard solutions of potassium ferrocyanide, phosphate/acetate buffers and representative natural phenols in a wide pH range (3.0 to 9.0). Among the natural phenols herein investigated, the highest sensitivity was observed for rutin, a powerful antioxidant widespread in functional foods and ubiquitous in the plant kingdom. The calibration curve for rutin performed at optimum pH (7.0) was linear in a broad concentration range, 1 to 120 µM (*r* = 0.99), showing detection limits of 0.4 µM. The optimized biomimetic sensor was also applied in total phenol determination in natural samples, exhibiting higher stability and sensitivity as well as distinct selectivity for antioxidant compounds.

## 1. Introduction

Laccases are oxidase enzymes constituted by dimeric or tetrameric glycoproteins with up to four copper atoms per monomer, which are distributed in their active site. They are very useful for technological development due to their ability to catalyze a wide range of redox processes including oxidation reactions for pollutant removal. These enzymes use oxygen as an electron acceptor and a variety of organic compounds as substrates such as monophenols and polyphenols [1,2,3,4,5]. Since oxidation occurs by reducing four oxygen electrons to water molecules, laccase activities are higher for organic compounds presenting a low redox potential, i.e., *orto* and *para* di-phenols [3,4,5,6,7].

Phenolic compounds are ubiquitous in the environment as phenolics are released to ecosystems from natural or anthropogenic sources [4,5,6]. The relevance of such compounds relates to their properties in terms of toxicity and environmental impacts [4,5,6,7] as well as antioxidant and/or biological activities [8,9,10]. Regardless of such positive or negative properties and characteristics, the need for monitoring and/or analytical determination of phenolic compounds is no longer a question, with a recent search for eco-friendly analytical methods for phenolic determination already in place [4,5,6,7,8,9,10,11,12,13,14,15,16,17,18,19,20,21].

**Figure 1:**
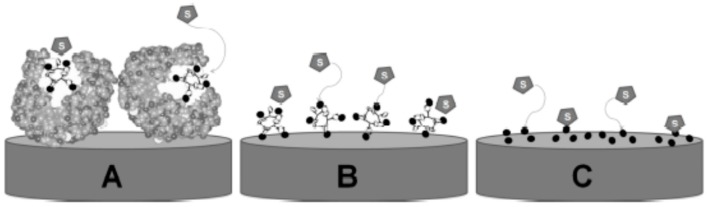
Schematic representation for substrate (S) access to: Enzymatic-based biosensor (**A**); Biomimetic systems based on mini-enzymes (**B**); and only on the prosthetic group (**C**).

Amperometric biosensors based on laccases combine the sensitivity of electrochemical transducers with the affinity/selectivity of laccases for phenols, resulting in very promising systems [6,13,14,15]. Nevertheless, the low stability of enzymes and other biological recognizing agents hampers their widespread long-term use and in situ practical applications [16,17,18,19,20]. To overcome this difficulty, the use of biomimetic systems (Figure 1) has been proposed as a promising alternative in the determination of different analytes [17,18,19,20,21].

In fact, a great number of transition metal compounds, akin to enzymes, are recognized by their catalytic properties. In the case of laccase enzymes, the redox catalytic site is centered in copper units, and thus their activity on phenol oxidation can be mimicked using copper-based materials [14,16,18,19,20,21]. Moreover, these systems can be also employed to improve the electrochemical and electrocatalytic properties of biosensors, leading to sensitivity and selectivity enhancement in amperometric biosensors. In fact, nanostructured metallic materials featuring improved electroactive areas can promote electron transfer kinetics and decrease the potential gap between the electrode and active site of the enzyme and substrate (analyte) [18,19,20,21]. Such a good analytical performance has been already reported for other biomimetic systems [15,16,17,18]. Enzyme-free biosensors based on metal oxides [19,20] and metal complexes [21] exhibited superior activity with respect to that of analogous enzymatic biosensors. In the case of multicopper oxidases such as laccases, a biomimetic system should ideally be based on copper complexes or oxides [20,21].

Based on these premises, this work aims to develop bio(mimetic) sensors based on modified carbon paste containing copper oxide and/or laccase, in order to establish a comparison between the systems and evaluate the electrocatalytic efficiency gain for the determination of phenolic compounds, as well as to estimate the antioxidant properties of certain natural samples.

## 2. Results and Discussion

**Figure 2:**
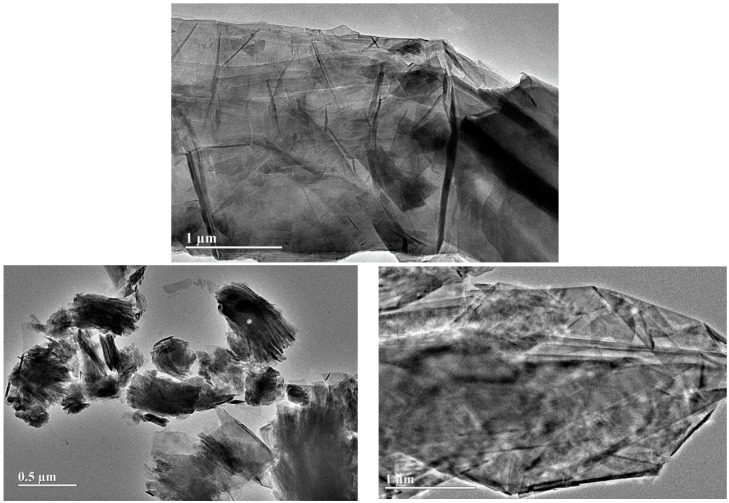
TEM image of S2 material (**bottom images**) as compared to the parent graphite powder (**top image**).

**Figure 3:**
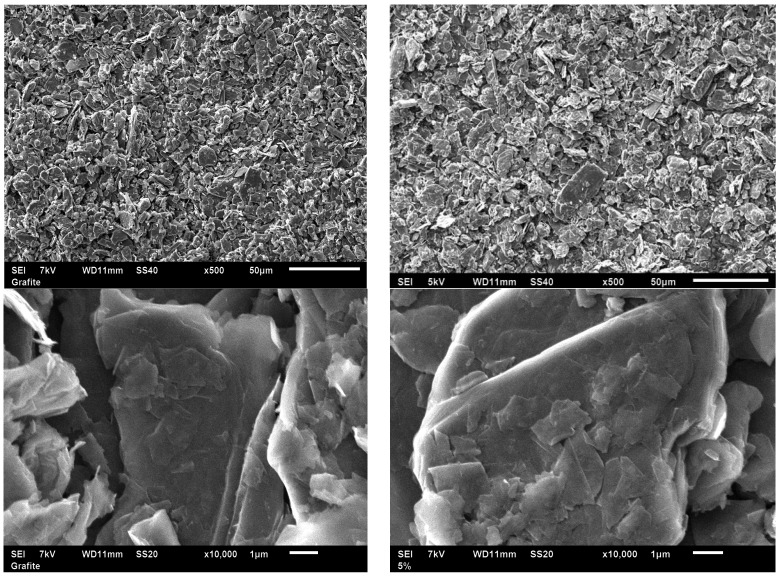
SEM images of graphite powder (**left images**) and S2 (**right images**) at different magnifications.

The modified graphite samples were isolated as powders and the impregnated copper was analyzed by ICP-OES. The result shows a different Cu content for material S1 (8.8 mg Cu/g graphite) and S2 (17.4 mg Cu/g graphite). These corresponded to a Cu content of 1.1% and 2.2% *w*/*w* for materials S1 and S2, respectively. The presence of copper oxide phases could not be confirmed by X-ray diffraction due to the very low copper concentration (ca. 1%–2%), but the proposed methodology has been previously reported to generate CuO as an active phase [12,13]. SEM and TEM images of the synthesized nanomaterials (Figure 2 and Figure 3) depict the generally nanosheet-type structure of the graphite support without any clear visualization of Cu nanoparticles (even for the higher-Cu-content S2 biosensor), in good agreement with the XRD results. SEM images also confirm the similarities and almost identical nature of the graphitic powder material as compared to S1 and S2 in terms of morphology and particle size (ca. 1–5 µm diameter for most particles). The low Cu loading did not significantly influence the structural and morphological properties of graphite but certainly its electrical properties (due to the presence of Cu, as confirmed by ICP/MS).

### 2.1. Electrochemical Properties

The electron transfer (ET) due to a electrochemically catalyzed redox process is related to the Faradaic current when the electrode overpotential is enough to overpass the thermodynamic and kinetic barriers [22]. This ET through the electrode material has been evaluated by using cyclic voltammetry in K_2_Fe(CN)_6_ solution. The Ferro/Ferri redox system is characterized by its reversibility and high ET kinetics. Therefore, a small gap would be expected between the oxidation and reduction overpotential for electrode materials presenting good conductive properties, ∆*E*_p_ (*E*_pa_ − *E*_pc_), as well as an equal peak currents ratio, *I*_pa_*/I*_pc_ = 1 [14,15]. Furthermore, the higher the conductivity, the higher the anodic (*I*_pa_) and cathodic (*I*_pc_) peak currents. On the other hand, the capacitive current increases according to the electrode resistance whereas the voltammetric profile presents the usual ascendant shape [14,15,16].

**Figure 4:**
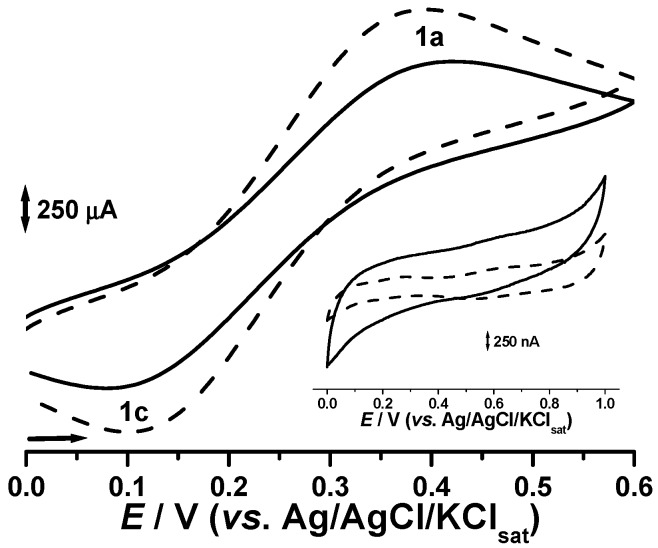
Cyclic voltammograms in 0.1 M KCl: 0.05 M potassium ferrocyanide. Inset: cyclic voltammograms in 0.1 M phosphate buffer, pH 6.0. Unmodified carbon paste (^___^) and CPS2 (- - -).

Figure 4 clearly demonstrates a significant improvement for supported copper oxide systems in terms of the reversibility of the Ferro/Ferri redox system (the ∆*E*_p_ was ca. 30 mV lower). Current intensities for both the anodic and cathodic currents are clearly higher for the potassium ferrocyanide probe solution, whereas the capacitive component results in a thinner and straighter voltammetric profile.

### 2.2. Electrocatalytic Properties

**Figure 5:**
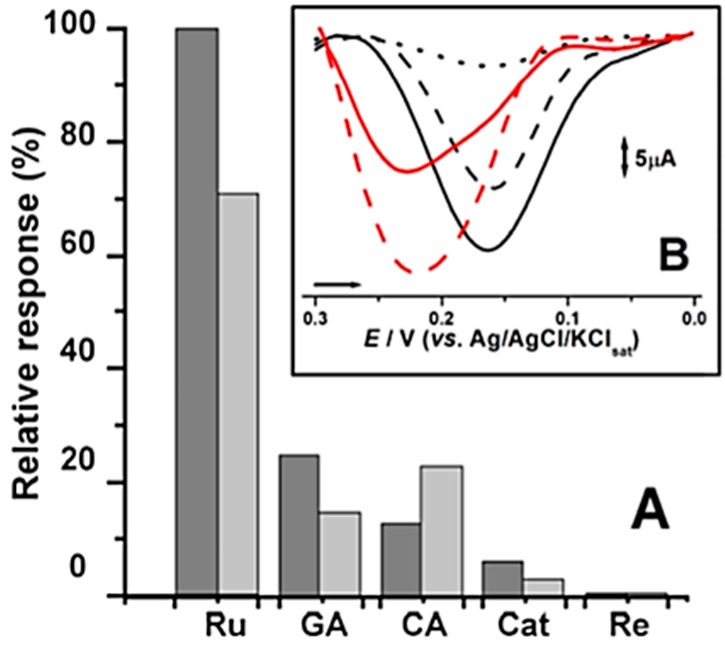
Electrodes responses in DP assays. (**A**) Relative response (%) obtained from DPV assays in pH 6.0 and 0.1 M PBS for 0.02 mM solutions of rutin (Ru); gallic acid (GA); caffeic acid (CA); catechol (Cat) and resorcinol (Re) at CPS2 (dark gray) and CPB (light gray); (**B**) DP voltammograms in 0.1 M PBS, pH 6.0 containing 0.1 mM catechol at different CP electrodes. unmodified CP (∙∙∙∙); CPS1 (- - -); CPS2 (**―**); CPB (―) and CPS2B (−−−).

In order to evaluate the improvement of electrical properties leading to electrocatalytic enhancement, the designed (bio)sensors CPS (enzyme-free sensor), CPB (laccase-based biosensor) and CPSB (laccase-based biosensor with copper oxide)were evaluated in the electroconversion of catechol as a substrate probe. The best response was achieved in systems containing a larger amount of copper oxide, namely CPS2 and CPS2B. Hence, both optimum sensors were subsequently tested against different natural phenol compounds (Figure 5).

Figure 5 clearly pointed out that enzyme-free sensor CPS1 exhibited a slightly superior electrocatalytic activity as compared to that of the laccase-based biosensor (CPB). An even more superior activity was also obtained for CPS2, containing a larger quantity of copper oxide. The overpotential required for the reduction ranged from 0.23 to 0.16 V which can be related to the acidic character of the enzyme. Such observed peak potentials are, anyhow, close to those reported for laccase biosensors under mild acidic conditions [4,5,6,7,11,12,13,14,15].

The analytical performance of the mixed system (CPSB), based on copper oxide and laccases, was also checked. A significant enhancement of electrocatalytic activity against catechol was again observed, proportional to the copper quantity in the material, but occurred at the same overpotential of CPB.

**Figure 6:**
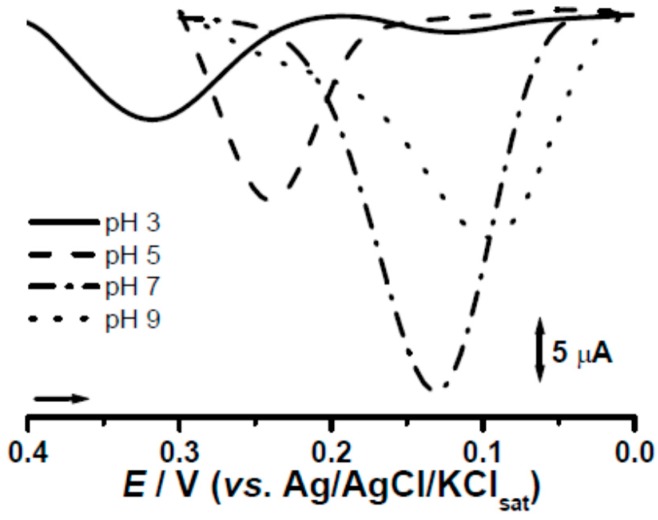
Differential pulse voltammograms for rutin at different pHs for CPS2.

Regarding the electrolyte conditions, pHs were carefully evaluated in the range of 3.0 to 9.0. The optimum response in the systems was achieved at pH 7.0 (Figure 6) for which all calibration curves and further studies were subsequently carried out.

**Figure 7:**
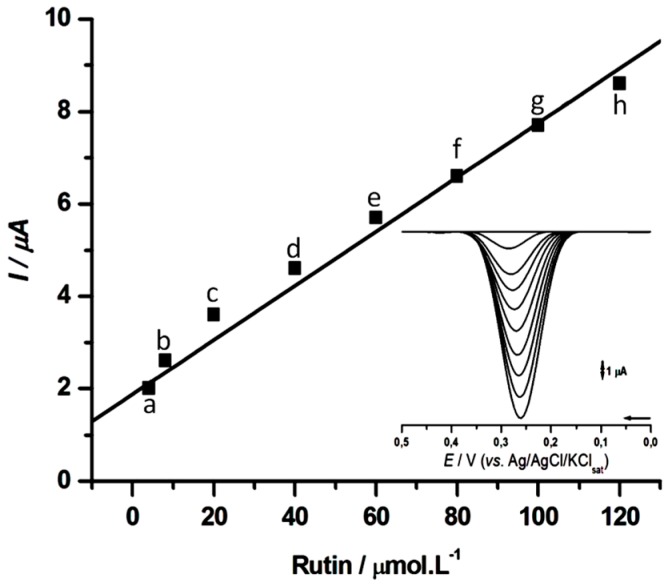
Calibration curve obtained from DPVs in 0.1 M PBS, pH 7.0, for increasing concentrations (a) 5, (b) 10, (c) 20, (d) 40, (e) 60, (f) 80, (g) 100, (h) 120 µM of rutin at CPS2.

The calibration curves for rutin were performed in 0.1 M PBS, pH 7.0, for CPS2 (Figure 7). The response was linear from 1 to 120 µM (*r* = 0.985), resulting in a linear equation, *I* = 1.876 + 0.059 C, in which *I* is the cathodic peak current (µA), and C the rutin concentration (µM). The observed detection limit (LoD) of 0.4 µM was comparatively lower to that reported for other laccase-based biosensors as summarized on Table 1.

### 2.3. Determination of the Antioxidant Properties of Natural Samples

Based on optimized studies, CPS2 was selected as an optimum biomimetic sensor and was subsequently employed to determine rutin and catechol equivalents in certain natural samples, namely dried extracts of red fruits and coffee. Results were further compared to traditional methods for radical scavenge assays (DPPH) and Folin Ciocalteu (FC) for total phenol content (TPC) evaluation. Results presented on Table 2 pointed out that rutin and catechol equivalents determined by CPS2 as a biomimetic sensor possessed a good correlation with the results obtained from FC total phenol content and DPPH radical scavenging assays. As expected, the rutin and catechol equivalents were proportional to the TPC results obtained by means of the FC assay, whereas the DPPH assay, expressed by the amount leading to 50% of decolonization, presented an inverse correlation. The linear coefficients were calculated and they varied from 0.9609 to 0.9995. These findings demonstrate that the proposed simple methodology can, in principle, be applicable to qualitatively/quantitatively evaluate antioxidant properties of phenolics present in food, pharmaceuticals and clinical routine analysis.

## 3. Material and Methods

### 3.1. Reagents and Samples

All reagents were of analytical grade. Graphite powder, copper chloride, potassium ferrocyanide and all phenolic standards were purchased from Sigma-Aldrich Chemical Co. (St. Louis, MO, USA). Mineral oil and the electrolyte buffer salts were purchased from Merck (Darmstadt, Germany).

Foodstuffs samples with known antioxidant activity were purchased from local markets and drugstores of the city of Goiânia, Goiás, Brazil.

Assay samples were prepared by sonication in ethanol:buffer (1:1) solution in order to achieve a 10% final concentration. Standards were prepared in suitable solvents in order to get stock solutions of 10 mM final concentration.

### 3.2. Preparation of Modified Graphite Powder

Copper oxide nanoparticles were deposited via conventional impregnation on a graphite surface (CuO-C) according to literature procedures [19]. Briefly, 10 g of graphite powder was activated by heating under reflux in 10 mL of 3 M HNO_3_:H_2_SO_4_ (1:1) solution under stirring (2 h). The obtained solid was filtered, washed with distilled water and dried overnight at 80 °C. The solid was then re-suspended in 30 mL of copper chloride solutions (2% and 5% *w*/*w*) and the mixture was stirred for an additional hour. The impregnation of copper oxide (CuO) on the carbon surface was achieved by co-precipitation by addition of KOH (10 mM):KNO_3_ (1 mM) solution at 90 °C. The solid was filtered and calcined at 300 °C for 2 h under nitrogen atmosphere.

The obtained CuO-C composites were characterized accordingly to the copper amount and owing to their potential use as conducting and electrocatalytic materials for sensing purposes (S1 and S2).

### 3.3. Copper Analysis on Modified Graphite Powder

The copper concentration in graphite powder samples was determined by Inductively Coupled Plasma Optical Emission Spectrometry (ICP-OES) in a PerkinElmer-model 7300 DV with Hydride Vapor Generator (PerkinElmer, Norwalk, CT, USA). Then the mixture was filtered, washed with deionized water and the solution analyzed for Cu.

X-ray diffraction measurements were performed on a Bruker D8 Discover diffractometer composed by copper anode coupled to a Johansson monochromator (Brucker Daltonics, Bremen, Germany). The samples were kept in rotation of 15 rpm during measurement to minimize the effects of preferred orientation.

### 3.4. Preparation of Laccase Extract

*P. sanguineus* (CCT-4518) fungus was maintained through periodic transfer at 4 °C on potato-dextrose-agar (PDA, HiMedia, Mumbai, India). Growth was carried out at 28 °C in the dark by pre-inoculating. The fungus growth was carried out in 50 mL of liquid medium containing 12.8 g·L^−1^ of malt extract (HiMedia), 0.005 g·L^−1^ CuSO_4_·H_2_O (Cromoline, São Paulo, Brazil) and 50 mg·L^−1^ 2.5-xilidine (Sigma-Aldrich Chemical Co.) in Erlenmeyer flasks (250 mL) with five agar disks (7 mm diameter) of mycelium grown on PDA. Cultures were incubated per 72 h in the dark at 28 °C under agitation (150 rpm) [23].

Partial purification of enzymatic extracts was conducted by filtering (14 µm, Frisenette ApS, Knebel, Denmark) of the mycelium. The supernatant was stored at 4 °C and this resulting crude extract was used for further biosensor development.

### 3.5. Preparation of (Bio)Sensors

Carbon paste sensors (CPS) were synthesized by mixing rigorously 75 mg of graphite, modified with ca. 1% (CPS1) and 2% (CPS2) of copper oxide in a mortar, with 25 mg of mineral oil (as agglutinating agent). Comparatively, carbon paste biosensors (CPB) were produced by adding optimized 250 µL amount of laccase crude extract (2019 U·L^−1^), to unmodified graphite or copper oxide graphite (CPS1 and CPS2), as described above. The resulting paste was mixed and left to dry at room temperature. Then 25 mg of mineral oil was then added to the dried powder and thoroughly mixed in a mortar leading to a homogeneous carbon paste.

An appropriate portion of the agglutinated carbon pastes were used to fulfill the cavity (2 mm diameter and 0.5 mm depth) of the support electrode.

The resulting sensors and biosensors are detailed in Table 3.

### 3.6. Electroanalytical Assays

Measurements were performed with the potentiostat/galvanostat μAutolab III^®^ (Metroh Autolab B.V., Utrecht, The Netherlands), integrated with GPES 4.9^®^ software (Eco-Chemie, Utrecht, The Netherlands), in a 5 mL electrochemical cell of three-electrode system, in which the carbon paste based (bio)sensors described in Table 1 (Ø = 2 mm), a Pt wire and Ag/AgCl/KCl_sat_, represented, the working, counter and reference electrode, respectively. The carbon paste was mechanically renewed every new analysis.

The experimental conditions for differential pulse voltammetry (DPV) were: pulse amplitude of 50 mV, pulse width of 0.5 s, sweep speed 10 mV·s^−1^ and scan range from 0.4 to 0 V. The experimental conditions for cyclic voltammetry (CV) included a sweep rate from 25 to 250 mV·s^−1^ and a scan range 0 to 1 V. All experiments were performed at room temperature (21 ± 1 °C) in triplicate (*n* = 3). The electrolyte solutions used were 0.1 M acetate buffer (pH 3 and 5) and 0.1 M phosphate buffer (pH 6 and 9).

The electrochemical behavior of the different electrode materials were evaluated by means of CV in buffer and 1 M potassium ferrocyanide probe. The electrocatalytical properties were evaluated by DPV using 1 mM catechol solution.

Data obtained in GPES 4.9^®^ software were treated with the purpose of improving the visualization and identification of peaks without introducing any artifacts. Plots of the voltammetric curves for final presentation in this study were drawn using Origin Pro 8^®^ software (Northampton, MA, USA).

### 3.7. Total Phenol and Radical Scavenging Assays

For the determination of total phenolic content, the adjusted Folin-Ciocalteu (FC) method [6,7,15] was selected and the results expressed by means of gallic acid equivalents (GAE), mg of GA in each mL of crude sample.

The radical scavenging activity assays were performed using stable DPPH (2,2-diphenyl-1-picrylhydrazyl), in accordance with very well established procedures [6,7,15], whereas the antioxidant activity was expressed as EC_50_, representing the amount (µL) of sample solution to produce 50% of decolorization of DPP• relative to the blank control. Both spectrometric traditional methods were analyzed in a 1 cm glassy cell length at room temperature.

Crude samples were prepared by infusion of 2 g of coffee soluble granules in 25 mL hot water or by sonicating 2 g of the red fruits dried extracts in 25 mL ethanol:water (1:1).

## 4. Conclusions

Synthesized copper oxide–modified graphite biomimetic sensors were proved to be high performance electrode materials for phenolic determination. These exhibited comparable (or even improved) analytical performance as compared to laccase-based biosensors. The enhancement in sensitivity found when using catechol as a model solution could be simply correlated to the amount of incorporated copper oxide, pointing to both improvements on the electroactive surface area as well as the electrocatalytic properties. Optimum response was achieved in neutral pH, consistent with phenol hydrogen atom transfer (HAT) oxidation mechanisms.

The proposed biomimetic sensor also exhibited a remarkable sensitivity and selectivity for rutin, in a broad linear concentration range, which is useful to determine antioxidant equivalents in natural products (with an excellent correlation between sensor results and the DPPH assay). We envisaged this simple methodology to be also potentially extended to the screening of new natural antioxidants present in various matrices that will be reported in due course.

## Figures and Tables

**Table 1:** Laccase-based (bio)sensors for phenol determination.

Biosensor	Phenolic Substrate	Linearity (µM)	LD (µM)	Reference
CPS2	Rutin	1 to 120	0.4	This work
	Catechol	10 to 600	2.0	
Laccase on CP	Catechol	20 to 700	4.5	[5]
Laccase entrapped in chitosan microparticles	Catechol	0.2 to 13	0.02	[11]
Laccase immobilized on polyethersulfone membrane	Rutin	1 to 10	<1	[13]
Laccase/DNA/chitosan CP	Catechol	38 to 140	12	[14]
Laccase/tyrosinase/titanium gel matrix	Catechol	0.2 to 23	0.13	[15]

**Table 2:** Total phenol and antioxidant assays obtained for natural samples.

Sample	CPS2		Antioxidant Assays	
	Rutin Eq	Catechol Eq	DPPH	FC-GA Eq
	(µM)	(mM)	EC50	mg/mL
Coffee (*N* = 3)	44.28 ± 1.5	1.03 ± 0.015	17.55 ± 1.5	0.55 ± 0.11
Acerola (*N* = 3)	35.47 ± 1.9	0.94 ± 0.019	23.02 ± 1.6	0.44 ± 0.11
Açai (*N* = 3)	37.90 ± 0.6	0.96 ± 0.006	21.73 ± 1.8	0.50 ± 0.11
Cranberry (*N* = 3)	54.57 ± 1.4	1.13 ± 0.014	14.29 ± 1.9	0.61 ± 0.11

**Table 3:** Developed carbon paste sensors and biosensors.

(Bio)Sensor	CuO-C *	Laccase (µL)
CP	0%	-
CPB	0%	250
CPS1	1%	-
CPS1B	1%	250
CPS2	2%	-
CPS2B	2%	250

* % (*w*/*w*) of CuO per graphite.
